# Interventions for improving outcomes in patients with multimorbidity in primary care and community setting: a systematic review

**DOI:** 10.1186/s13643-021-01817-z

**Published:** 2021-10-20

**Authors:** Susan M. Smith, Emma Wallace, Barbara Clyne, Fiona Boland, Martin Fortin

**Affiliations:** 1 Department of General Practice and HRB Centre for Primary Care Research, Royal College of Surgeons, 123 St Stephens Green, Dublin 2, Ireland; 2 Data Science Centre and HRB Centre for Primary Care Research, Royal College of Surgeons, 123 St Stephens Green, Dublin 2, Ireland; 3 Department of Family Medicine and Emergency Medicine, Université de Sherbrooke, Quebec, Canada

## Abstract

**Background:**

Multimorbidity, defined as the co-existence of two or more chronic conditions, presents significant challenges to patients, healthcare providers and health systems. Despite this, there is ongoing uncertainty about the most effective ways to manage patients with multimorbidity. This review updated and narrowed the focus of a previous Cochrane review and aimed to determine the effectiveness of interventions designed to improve outcomes in people with multimorbidity in primary care and community settings, compared to usual care.

**Methods:**

We searched eight databases and two trials registers up to 9 September 2019. Two review authors independently screened potentially eligible titles and selected studies, extracted data, evaluated study quality and judged the certainty of the evidence (GRADE). Interventions were grouped by their predominant focus into care-coordination/self-management support, self-management support and medicines management. Main outcomes were health-related quality of life (HRQoL) and mental health. Meta-analyses were conducted, where possible, but the synthesis was predominantly narrative.

**Results:**

We included 16 RCTs with 4753 participants, the majority being older adults with at least three conditions. There were eight care-coordination/self-management support studies, four self-management support studies and four medicines management studies. There was little or no evidence of an effect on primary outcomes of HRQoL (MD 0.03, 95% CI −0.01 to 0.07, *I*^2^ = 39%) and mental health or on secondary outcomes with a small number of studies reporting that care coordination may improve patient experience of care and self-management support may improve patient health behaviours. Overall, the certainty of the evidence was graded as low due to significant variation in study participants and interventions.

**Conclusions:**

There are remaining uncertainties about the effectiveness of interventions for people with multimorbidity, despite the growing number of RCTs conducted in this area. Our findings suggest that future research should consider patient experience of care, optimising medicines management and targeted patient health behaviours such as exercise.

**Supplementary Information:**

The online version contains supplementary material available at 10.1186/s13643-021-01817-z.

## Background

There is now greater recognition of the impact of living with multiple chronic conditions, defined as multimorbidity and of the importance of improving outcomes for individuals affected [1–3]. Individuals with multimorbidity are more likely to die prematurely, be admitted to hospital and have longer hospital stays [4, 5]. They have poorer quality of life, loss of physical functioning, and are more likely to suffer from psychological stress [6–9]. The negative impact of multimorbidity is higher in the most disadvantaged communities with earlier onset and more complex combinations of mental and physical health conditions [10, 11]. Medicines management is often complex, resulting in polypharmacy with its attendant risks of drug interactions and adverse drug events [12, 13]. Patients must also attend multiple appointments with different healthcare providers and adhere to lifestyle recommendations. This adds to complexity and can sometimes lead to confusion with multiple treatments and guidance adding to treatment burden for patients [14]. Fragmentation of care is a significant problem for this group, resulting from the involvement of both primary care and multiple specialists who may not be communicating with each other effectively [15]. Clinical guidelines that address multimorbidity and related areas such as polypharmacy have emphasised the need for good quality evidence from primary studies [13, 16].

Given the challenge of managing people with multimorbidity, potential interventions are likely to be complex and multifaceted. The previous Cochrane review of interventions for multimorbidity [17] incorporated studies targeting both multimorbidity and comorbidity but as evidence evolves a distinction needs to be made between these two concepts. Interventions for comorbidity studies include specific groups of patients and can be designed to target the index and comorbid conditions, for example diabetes and comorbid depression. On the other hand, interventions for multimorbidity need to have a more generic focus that will work across a broad range of conditions. These distinctions are important in the context of developing and evaluating effective interventions for multimorbidity and considering their generalisability [18]. This systematic review updated and narrowed the focus of a previous Cochrane review and aimed to determine the effectiveness of interventions designed to improve outcomes in people with multimorbidity in primary care and community settings.

## Methods

This systematic review is an update and adaptation of a previous Cochrane review, published in 2016 with searches up to September 2015 and which had included both multimorbidity and comorbidity [17]. The review is reported using the Preferred Reporting Items for Systematic Reviews and Meta-Analyses (PRISMA) guidelines for systematic reviews [19].

### Search strategy

We searched MEDLINE, EMBASE, CINAHL, The Cochrane Library’s five databases and two trials registers up to 9 September 2019. We also searched grey literature, in particular, the comprehensive database of the International Research Community in Multimorbidity and abstracts from annual meetings of the Society of Academic Primary Care and the North American Primary Care Research Group. We also consulted experts in the field for completed or ongoing studies, over a number of years. Search strategies are available in Additional file 1: Search Strategy.

### Inclusion criteria

Study designs eligible for inclusion were randomised controlled trials (RCTs), non-randomised clinical trials (nRCTs), controlled before-after studies (CBAs), and interrupted time series analyses (ITS), meeting Cochrane Effective Practice and Organisation of Care (EPOC) quality criteria [20]. We included studies of adults with multimorbidity receiving care in a primary or community care setting. We adopted the most widely used definition of multimorbidity, that is, the co-existence of multiple chronic conditions in the same individual, usually defined as two or more conditions. We used the WHO definition of chronic disease, which is ‘health problems that require ongoing management over a period of years or decades’ [21]. Studies in which inclusion was based on comorbidity with a specific index condition [22] or only the age of participants (e.g. older patients) were excluded. We also excluded professional educational interventions where no care was delivered to an identified group of people with multimorbidity.

We included any type of intervention based in primary care and community settings that was specifically directed towards a group of people defined as having multimorbidity. Primary healthcare was defined as providing ‘integrated, easy to access, healthcare services by clinicians who are accountable for addressing a large majority of personal healthcare needs, developing a sustained and continuous relationship with patients, and practising in the context of family and community’ [23]. We anticipated that all interventions would be multifaceted given the nature of multimorbidity. We considered and reported complex interventions using the TIDIER checklist [24]. We categorised interventions based on their predominant intervention focus into the following groupings: (i) care coordination plus support for self-management; (ii) support for self-management, and (iii) medicines management. The comparison was usual primary healthcare as provided in that setting.

### Review processes

One author undertook an initial screen of abstracts to remove those clearly ineligible. Two authors (SS, EW) then independently screened remaining abstracts and identified full texts for screening, screened full texts and selected studies for inclusion. No automated tools were used in the process. Two authors (SS, EW) undertook data abstraction and cross-checked data abstraction forms. Disagreements about data abstraction were resolved by consensus between the authors. If data were missing, we contacted authors and have reported this where applicable. Two authors assessed and cross-checked the risk of bias in all included studies using Cochrane criteria (SMS and EW or BC), including allocation (sequence generation and concealment); baseline characteristics; incomplete outcome data; contamination; blinding; selective outcome reporting and other potential sources of bias. We assessed the certainty of the evidence for health-related quality of life (HRQoL), mental health, clinical, psychosocial, health service utilisation, medicines and provider behaviour outcomes using the Grading of Recommendations Assessment, Development and Evaluation (GRADE) criteria including risk of bias, consistency of effect, imprecision, indirectness and other potential criteria such as publication bias [25].

### Outcomes

We based our main outcomes for this review on the core outcome set for multimorbidity [26] and these were health-related quality of life (HRQoL) and mental health outcomes. Additional outcomes included clinical outcomes, other psychosocial outcomes such as self-efficacy, health behaviours, healthcare utilisation, medicines outcomes, provider behaviour, including quality of care, patient satisfaction, harms and economic outcomes. Where data from multiple timepoints was reported, we extracted the data from the designated study end-point.

### Analysis

Due to the clinical heterogeneity relating to the wide variation in participants, interventions and outcomes assessed, the main synthesis of the results is narrative. This decision was made by all authors on reviewing the range of participants and interventions as presented in the Table of Included studies. We undertook meta-analysis where it was appropriate to combine studies in terms of participants, interventions or outcomes and in these cases we used risk ratios or mean differences in the synthesis. We did not impute any missing data, and clustering in original studies was already incorporated in included study estimates within the meta-analyses. Meta-analysis was undertaken in the Revman software for the main outcome HRQoL using a random-effects model with generic inverse variance, which incorporates cluster effects within estimates for each included study. We also conducted a random effects meta-analysis of mean difference in two studies for the additional outcome of self-efficacy using.

## Results

### Results of the search

**Fig. 1:**
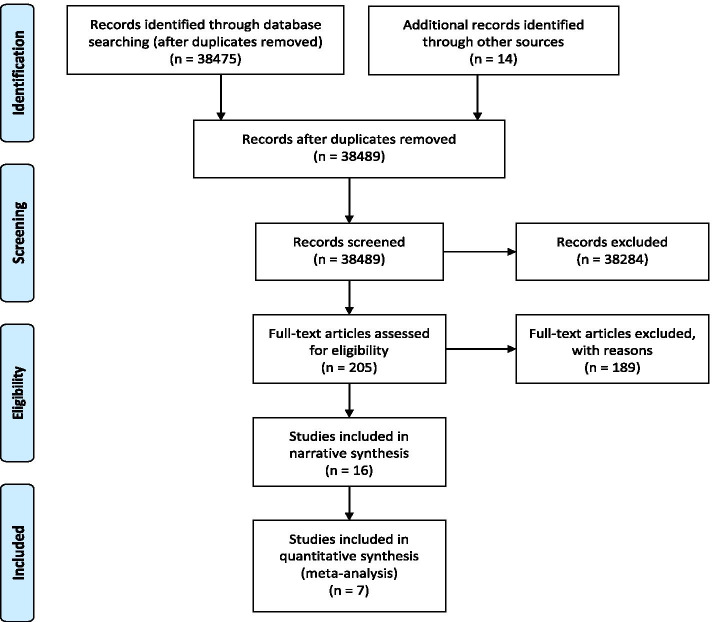
Flow diagram of studies screened

From a total of 38,489 original citations (after duplicates were removed) (Fig. 1), 205 full texts were reviewed. Of these, 189 articles were excluded and a total of 16 RCTs were included. Sixteen studies contributed data for inclusion in the narrative synthesis and 7 provided data for meta-analyses.

### Included studies and participants

A total of 16 RCTs with 4753 participants were included. Eight had a parallel design [27–34], and eight had a cluster design [35–42], with one of these having a cluster stepped wedge design [37]. Intervention duration varied from 6 weeks to 18 months, with the majority lasting 6 to 12 months. Most studies collected follow-up data at intervention completion. The studies were conducted in Germany (*n* = 4), UK (*n* = 3), USA (*n* = 3), Canada (*n* = 2), Ireland (*n* = 2) and one study each in Spain and Australia. All studies were funded publicly by government agencies or through charitable or university foundations. The definition of multimorbidity varied across studies though all used some additional measure of complexity beyond the standard multimorbidity definition of 2 or more conditions. These included higher numbers of conditions or additional factors such as high health service use or polypharmacy. The mean number of conditions in patients in the 14 studies that reported this, ranged from 3 to 12.7 conditions (see Table 1), suggesting that included studies were targeting those with more complex multimorbidity. Whilst six of the 16 studies targeted older patients, the mean age of included patients ranged from 50 to 80.5 years with 10 of the 16 included studies having participants with a mean age > 70 years indicating that most studies included older patients (see Table 1).

**Table 1:** Characteristics of included studies

Study ID Design Country	Study participants Duration and follow-up	Intervention aim, elements and comparison TIDIER checklist Why: Aim What: procedures and materials Where; When and how much Who provided	Primary outcome
**Care coordination or planning and support for self-management**			
Boult 2011 [35] RCT USA	904 adults > 65, multimorbid and high service use, mean age 77 and mean 4.3 conditions Intervention 18 months, follow-up at 6 and 18 months	Aim: to measure the effect of guided care teams on multimorbid older patients’ use of health services Guided Care (GC): Enhanced multidisciplinary team providing self-management support Home assessments and coordination of care by GC nurses with monthly monitoring over 18 months Patient care plans and educational materials Providers: Eight primary care systems, 14 GC nurses, 49 primary care physicians and managing 50‑60 patients, training of nurse managers Comparison: Usual care	Health service use
Contant 2019 [27] RCT (Fortin 2016) Canada (secondary analysis of multimorbidity sub-group)	281 patients 18 to 75 years of age with at least 3 of the following chronic conditions diabetes, cardiovascular disease, COPD, asthma, tobacco smoking, obesity and hyperlipidemia, mean age 53.4 and mean 5.4 conditions Intervention 3 months, follow-up immediately post intervention	Aim: To analyse the effect of a multidisciplinary self-management intervention amongst patients with multimorbidity. PR1MaC: Initial nurse evaluation with design of individualised intervention plan in collaboration with the patient, based on their objectives; and adaptable over time. Printed information and other educational material for patients At least 3 individual encounters with trained chronic disease prevention and management (CDPM) professionals over 3 months Providers: Four primary care clinics with doctors working together in group practices. Could include encounters with 1 or more CDPM professionals in the following disciplines: nursing, physical activity, nutrition, respiratory therapy and smoking cessation therapy Comparison: Usual care	Self-management (Health Education Impact Questionnaire (heiQ))
Gonzalez Ortega 2017 [30] RCT Spain	161 adults with significant chronic disease in 3 or more organ systems; mean age 80.5, mean 3.9 conditions, mean 8.4 medications. Intervention duration 6 months with immediate follow up at intervention completion	Aim: To evaluate the impact that adding a telephone coaching intervention by a family physician to usual care has on reducing resource consumption and improving health status, caregiver burden and quality of life amongst complex chronic patients compared with usual care. Telephone coaching and support for self-management by an intervention primary care physician (PCP). Patients had initial face-to-face meeting in their home or in the clinic and were then phoned twice a month over 6 months. Calls addressed symptoms, medications, social contexts and support for self-management. The PCP also reviewed the patients’ record and added notes regarding the calls. Providers: Three Primary Care teams. One independent intervention PCP. Comparison: Usual are from own PCP	Emergency admissions
Hochhalter 2010 [31] RCT USA	79 adults aged > 65, with ≥ two of seven chronic conditions; Mean age 74 and mean 3.6 conditions Intervention three months, follow-up 3 months after intervention	Aim: to test the efficacy of a patient engagement intervention for older adults with multiple chronic illnesses. Patient engagement intervention Led by ‘coaches’ with focus on making most of healthcare, supporting self-management. Checklists and protocols for coaches to follow during the workshop and calls. Two-hour workshop and two telephone calls a week before and a week after a medical appointment. Intervention was designed to prepare patients for appointments, to communicate effectively during appointments and follow through on care plans. Providers: Large Internal Medicine clinic. Coaches (professional qualifications and number coaches not reported) Comparison: 1. Attention control: 2-h workshop on safety issues and calls before and after a naturally occurring medical encounter. 2. Usual care	Self-management (patient activation measure)
Mercer 2016 [38] Cluster RCT (exploratory) Scotland	142 patients from 8 general practices in areas of deprivation, with ≥ two long term conditions; mean of 4.9 conditions, and mean age 52 Intervention duration 12 months with data collection at 6 months and at intervention completion	Aim: to evaluate a whole-system primary care-based complex intervention, called CARE Plus, to improve quality of life in multimorbid patients living in areas of very high deprivation. CarePlus: Primary care-based whole-system intervention Structured extended GP consultations and relationship continuity Practitioner support and training Patient self-management support with patient support materials Providers: Eight general practices in the most deprived parts of Glasgow Comparison: Usual GP care	Health-related quality of life (EQ-5D-5L) and well- being (W-BQ12)
Salisbury 2018 [40] Cluster RCT UK	1546 patients from 35 practice aged 18 years or older, with ≥ 3 chronic condition, based on 17 chronic conditions in Quality and Outcomes Framework; mean age 71 years, mean 3 conditions Intervention duration 15 months and outcomes measured at 9 and 15 months	Aim: The aim of this study was to implement and assess the effectiveness of a new approach to managing patients with multimorbidity in primary care. 3D intervention based on patient-centred care with focus on continuity, coordination, and efficiency of care with 6-monthly comprehensive multidisciplinary review (nurse, pharmacist and physician/GP) with extended appointments if requested. IT support to facilitate identification of patients, recall and 3D templates Printed care plans to support shared decision making Practice training: 2 half-days Practice supports: nominated practice 3D champion, automated monthly feedback compared to peers and financial incentives for completed reviews (GBP 30 per review). Providers: 33 general practices with named GP, practice nurse and pharmacists (who may or may not have worked with the practice previously) Comparison: Usual GP care	Health-related quality of life (EQ-5D-5L)
Schafer 2018 [41] Cluster RCT Germany	650 patients from 55 general practices with ≥ 3 conditions; mean age 73.5, mean 8.5 chronic conditions, mean 7 medications. Intervention duration: 12 months with final data collection at intervention completion	Aim: To determine if patient-centred communication leads to a reduction in the number of medications taken without reducing health-related quality of life. Multicare AGENDA: Patient-centred communication GP Training: 3 sessions lasting 4 h on narrative based patient-doctor dialogues Three 30 min ‘talks’ between GP and patients over 12 months: 1. Focus on patient priorities (including non-medical) 2. Medication review 3. Review previous goals and considered goal attainment at end of 12 months Providers: 55 general practices Comparison: Usual care with wait-list control	Number medications and Health-related quality of life (EQ-5D)
Sommers 2000 [42] RCT USA	543 adults aged > 65 with at least two conditions; mean age 77.5, mean number conditions not reported Intervention 18 months, follow-up 12 months after intervention	Aim: To examine the impact of an interdisciplinary, collaborative, practice intervention for community dwelling seniors with chronic illnesses Senior Care Connections Enhanced multidisciplinary teams with 2 months immersion in primary care practice for the nurses and social workers before intervention commenced Initial home assessment by the nurse or social worker to gather data on patient concerns Team then met and drafted risk reduction care plans and support for self-management to discuss with patients and family members Nurse or social worker monitored patients every 6 weeks between primary care physicians (PCP visits) either in home, in clinic or by phone Monthly team meetings to discuss patient progress with training and ongoing support for nurses and social workers. Providers: 18 PCPs working in 9 teams with a full-time nurse with geriatrics training and half-time social worker per team Comparison: Usual care	Health service use and self-rated health
**Support for self-management**			
Eakin 2007 [28] RCT USA (multimorbidity sub-group data from authors)	175 adults with ≥ 2 conditions (of 14 conditions listed), mean age 50; mean conditions not reported Intervention 16 weeks, follow-up 6 months after intervention	Aims: To address multiple risk factors in patients targeting low-income, largely Spanish speaking patients with multiple chronic conditions Self-management support, diet, and exercise intervention based on chronic care model Patient education materials with three tailored newsletters and linkage to local services Two structured visits (home or clinic) lasting 60‑90 min and two follow up telephone contacts over 16 weeks Providers: An experienced bilingual health educator working in a community health centre providing primary healthcare services to low-income and medically underserved individuals Comparison: usual care plus a guide to local services and three newsletters	Dietary behaviour and physical activity
Garvey 2015 [29] RCT Ireland	50 participants with ≥ 2 chronic conditions and 4 repeat medications, median age 66, median 4.5 conditions Intervention duration: 6 weeks with 2-week post intervention follow-up	Aim: to address the challenges of living with multimorbidity in a primary care setting. OPTIMAL, occupational therapy (OT) led self-management support course Focus on goal setting and prioritisation Peer support through group meetings Weekly meetings in local health centre over 6 weeks, meeting duration 2.5 h Providers: Three primary care centre. Primary care OTs in each centre led the programme with input from physiotherapist and pharmacist for one session each. Training and intervention manual for OT providers, provided by the research team. Comparison: Wait-list control. Received usual care whilst waiting.	Activity participation (Frenchay Activities Index)
O’Toole 2020 [33] RCT Ireland	149 patients aged over 18, ≥ 2 conditions and 4 regular medicines. Mean age 65 years, mean number 4.5 conditions and mean 9 repeat medicines Intervention duration: 6 weeks with immediate post intervention (primary outcomes only) and 6 months follow-up	Aim: To evaluate the effectiveness of a group based, 6-week, occupational therapy led self-management support programme (OPTIMAL) for patients with multimorbidity and test the sustainability of its effect over time. OPTIMAL, occupational therapy (OT) led self-management support course Focus on goal setting and prioritisation Peer support through group meetings Weekly meetings in local health centre over 6 weeks, meeting duration 2.5 h Providers: Eight Primary care Centres. Primary care OTs led the programme with one session each from physiotherapist and pharmacist. Training and intervention manual for OTs, provided by the research team. Comparison: Wait-list control. Received usual care whilst waiting.	Health-related quality of life (EQ5D) and Activity Participation (Frenchay Activities Index)
Reed 2018 [34] RCT Australia	254 adults aged over 60 years with ≥ 2 conditions and neutral or poor self-rated health; mean age not reported, approx. 50% > 75 years, mean 4.5 conditions Intervention duration: 6 months with immediate follow-up	Aim: To determine whether a clinician-led chronic disease self-management support (CDSMS) programme improves the overall self-rated health level of older Australians with multiple chronic health conditions. Clinician-led CDSMS Programme which included goal setting and the development of individualised care plans, based on the Flinders CDSMS programme. Delivered by nurses or psychologists in the patients’ home, 3 home visits with 4 follow up phone calls over 6 months, delivered independently of GP care. Mentoring of clinicians by trained accreditors. Providers: Trained nurses and psychologists, mentor supervising them Comparison: Attention control - same number of visits to the study clinicians but did not receive the CDSMS programme	Self-rated Health
**Medicines management**			
Jager 2017 [36] Cluster RCT Germany	273 patients from 22 practices, aged >50 years, with at least 3 chronic diseases, more than 4 drugs, and at high risk for medication-related events; mean age 72.2, mean conditions 5.7 Intervention duration 9 months; follow-up at intervention completion	Aim: to assess the effect of a tailored programme to improve the implementation of three important processes of care for this patient group: (a) structured medication counselling including brown bag reviews, (b) the use of medication lists, and (c) structured medication reviews to reduce potentially inappropriate medication. PomP: A tailored medicines management programme Training and resources for general practitioners (GPs) and medical assistants: 4-h workshop Patients: educational materials, electronic information tool and reminders for patients Implementation action plans for each GP practice with focus on three priority actions for medicines management and consideration of patient preferences Providers: 22 GPs from 18 practices of 66 GP Quality Circles, mean 4.6 medical assistants per practice Comparison: Usual care plus GPs informed of prescribing targets and aware of which patients identified for the trial as high risk	Summary score of 10 prescribing indicators
Koberlein Neu 2016 [37] cRCT (stepped wedge design) Germany	162 adults age ≥ 65 years, with ≥ 3 chronic disorders affecting two different organ systems, at least one cardiovascular disease, at least one visit to the PCP in each of the preceding three-month intervals, five or more long-term medicines, mean age 76.8, mean number conditions 12.7, mean number medications 9.4 Intervention duration 15 months, variable intervention exposure based on stepped wedge design. Data extracted for first phase of 3 months when was intervention vs control and no variation in exposure	Aim: To evaluate the effectiveness of interprofessional medication management for elderly multimorbid patients WESTGEM intervention: Comprehensive medication management Medication management with primary care physicians (PCPs) who sent e-information to home care specialists Care provided by home-care specialists using case management, conducting a home visit and assessment and communicating this to a pharmacist who undertook a medicines review and made recommendations. PCPs then responsible for delivering recommendations Providers: 12 PCPs and attached home care specialists, pharmacist (number not reported) Comparison: Usual care with their PCP	Quality of medication therapy (MAI score)
Krska 2001 [32] RCT UK	332 adults aged ≥ 65 with ≥ 2 conditions and on ≥ 4 medicines; mean age 75 and mean 3.9 conditions Intervention three months, follow-up three months after drug review	Aim: To evaluate the effects of pharmacist-led medication reviews in elderly patients taking multiple medications Clinical pharmacist conducted a home visit with patients and created a pharmaceutical patient care plan, which was then entered in to the patient’s record and implemented by practice team Providers: Clinical pharmacist, General Practitioners (numbers not reported) Comparison: Usual care and had review of drug therapy by pharmacist but no pharmaceutical care plan implemented	Pharmaceutical care issues
Muth 2018 [16] Cluster RCT Germany	505 cognitively intact patients from 20 general practices, ≥ 60 years, ≥ 3 chronic conditions, ≥ 5 long-term medicines, mean age 72, Charlson score 3.1; CIRS score 7.7 Intervention duration: Intervention delivered over two sessions (HCA and then GP) sessions, lasting 35‑45 min each, follow-up at 6 and 9 months	Aim: to improve the appropriateness of medication in older patients with multimorbidity in general practice. PRIMUM: Prioritising Multimedication in Multimorbidity Pre-intervention training of 90‑120 min for healthcare assistant (HCA) and GP. HCA conducted a checklist-based interview with patients on medication-related problems and a brown bag review to reconcile their medications. HCA entered details into the computerised decision support system (CDSS) GP undertook a review assisted by the CDSS and optimised medication, discussed it with patients and adjusted it accordingly. Providers: 72 general practices and had to have HCA with access to internet Comparison: Usual care but the control practice teams also received the GP guidelines for ambulatory geriatric care to harmonise usual care in both groups	Medication Appropriateness Index (MAI)

### Description of interventions and comparators

The interventions were all multifaceted (Table 1). Few studies specifically reported patient involvement in intervention design though this was becoming more common in later studies [38, 40]. Studies were grouped into three broad groupings of care-coordination plus self-management support (CC/SMS), self-management support (SMS) and medicines management. Eight of the 16 included studies examined CC/SMS type interventions and involved multifaceted interventions that targeted the coordination of care, healthcare providers and also provided self-management support for patients [27, 30, 31, 35, 38, 40–42]. Four studies reported on SMS interventions that did not have a clear link to the patients’ healthcare provision [28, 29, 33, 34]. Three of these were group-based programmes based on the Chronic Disease Self-Management Support programme and the fourth involved health promoters working from community clinics to provide individual self-management support [28]. Four studies focused primarily on medicines management but specifically targeted patients with multimorbidity [32, 36, 37, 39]. In the majority of included studies, the comparator was usual primary healthcare.

### Risk of bias and certainty of the evidence

**Fig. 2:**
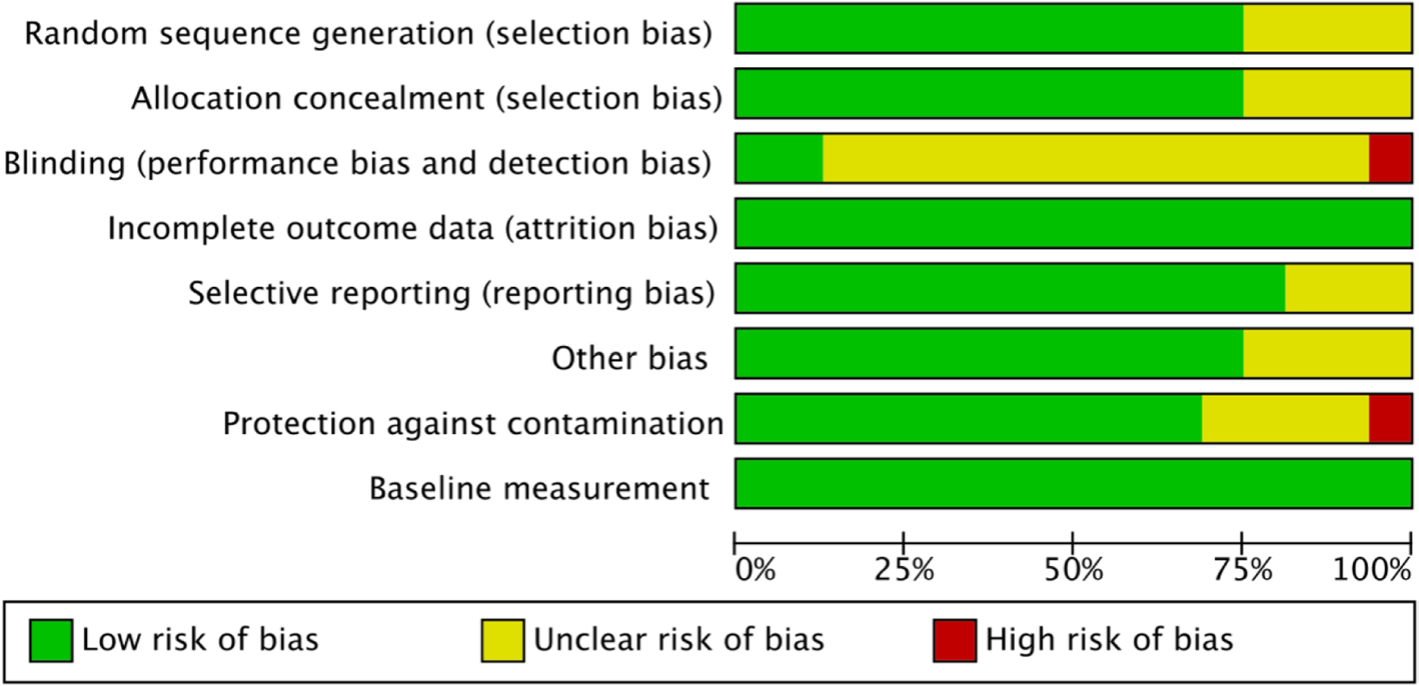
Risk of bias across included studies

The studies were all RCTs and overall there was low or unclear risk of bias with only one study having a high risk of bias in two of the eight risk of bias domains, blinding and protection against contamination [32] (see Fig. 2). The most common issue leading to a judgement of unclear risk of bias was lack of clarity around blinding, which can be a challenge in these types of interventions. The risk of bias for individual studies is presented in Additional file 2: Figure 1.

### Certainty of the evidence

In general, whilst all the included studies were RCTs, the main concerns related to inconsistency and imprecision. For all intervention types, we downgraded the evidence for all outcomes to low certainty due to serious concerns about inconsistency and imprecision (see Additional file 3: Grade Working Sheets). This reflects the clinical heterogeneity of participants, interventions and outcomes assessed and the likelihood that future studies may change our review findings.

### Effects of interventions

The effects of intervention on the main and additional outcomes are presented in Table 2.

**Table 2:** Outcomes and results

Study ID	Primary outcomes: Results	Secondary outcomes: Results
**Care-coordination/self-management support studies**		
Boult 2011 [35] RCT USA	Primary outcome: Health service use Adjusted ratio of service use: hospital 30 day readmissions 1.01 (95% CI 0.83 to 1.23); hospital days 0.79 (0.53 to 1.16); skilled nursing facility admissions 1.00 (0.77 to 1.30); skilled nursing facilities days 0.92 (0.6 to 1.4); emergency department visits 0.84 (0.48 to 1.47); primary care visits 1.04 (0.81 to 1.34); speciality care visits 1.02 (0.91 to 1.14); home healthcare episodes 1.07 (0.93 to 1.23)	PACIC (Patient Assessment of Chronic Illness Care) score at 18 months adjusted mean difference (aMD) 0.2 95% CI 0.07 to 0.33, *p* = 0.002 Satisfaction: no difference between groups Provider satisfaction with care mixed effects
Contant 2019 [27] (Fortin 2016) RCT Canada	Primary outcome: Self-management using the Health Education Impact Questionnaire (heiQ) 8 domains The intervention group showed improvement in 4 of the 8 heiQ domains in multivariate analysis: These four domains were: health-directed behaviour: OR 1.98, 95% CI 1.07 to 3.66, *p* −0.03; constructive attitudes and approaches: 3.92, 95% CI 1.73 to 8.89, *p* = 0.001; skill and technique acquisition OR 2.48, 95% CI 1.32 to 4.65, *p* = 0.005; health service navigation OR 2.73, 95% CI 1.2 to 6.22, *p* = 0.02. There were no significant improvements in positive and active engagement in life, emotional well-being, self-monitoring and insight and social integration and support.	Secondary outcomes were not reported in this secondary data-analysis study of Fortin 2016
Gonzalez Ortega 2017 [30] RCT Spain	Primary outcome: Emergency admissions After 6 months, urgent visits per patient decreased in intervention 1.27 baseline versus 0.89 follow-up, *p* = 0.091 and control 1.06 baseline versus 0.86 follow-up, *p* = 0.422, mean difference 0.18 [95% CI −0.48 to 0.84].	HRQoL SF12 significant effect on physical component score (aMD −4.71, 95% CI −9.03 to −0.41, *p* = 0.02) but no effect on the mental component score (aMD 2.6, 95% CI −3.9 to 9.11, *p* = 0.42) No significant effect on clinic visits; Charlson score; Function (Barthel); HRQoL; Cognitive status (Pfeiffer test); Pressure Ulcer risk (Norton scale); Social risk (Gijon Test); Caregiver Burden (Zarit test); chronic treatment (number of repeat medicines) or resource use (direct costs)
Hochhalter 2010 [31] RCT USA	Primary outcome: Patient activation measure (PAM). PAM Intervention 66.8 (18.5) vs Control 66.2 (13), no significant difference, all groups had significant improvement from baseline	Significant improvement in self-efficacy compared to usual care (but attention control group also had a significant improvement). No difference in total unhealthy days and self-rated health
Mercer 2016 [38] Cluster RCT (exploratory) Scotland	Primary outcomes: Health-related quality of life (EQ-5D-5L) and well-being (W-BQ12) EQ5D Index scores: 0.06 (95% CI −0.02 to 0.14, *p* = 0.15) EQ-5D-5L area under the curve over the 12 months was higher in the CARE Plus group (*p* = 0.002). CARE Plus significantly improved one domain of well-being (negative well-being), with an effect size of 0.33 (95% confidence interval [CI] 0.11–0.55) at 12 months (*p* = 0.0036). Positive well-being, energy, and general well-being (the combined score of the three components) were not significantly influenced by the intervention at 12 months.	No significant difference in anxiety and depression (HADS); self-efficacy self-esteem and medications Cost Effectiveness Analysis: Within-trial cost-utility analysis based on the EQ-5D-5L utility scores, and on health service utilisation: Adjusted mean difference in cost of GBP929 (95% CI 86 to 1788) per patient Gain in QALY 0.076 (95% CI 0.028‑0.124) Cost effectiveness ratio (CER) GBP12,224 per QALY
Salisbury 2018 [40] Cluster RCT UK	Primary outcome: HRQoL (EQ-5D-5L) No difference between groups with EQ-5D-5L aMD 0·00, 95% CI –0.02 to 0.02; *p* = 0·93.	PACIC score: aMD 0.29 (95% CI 0.16 to 0.41) Continuity of care score: adj MD 0.081; 95% CI 0.02 to 0.13 Mean Consultation and Relational Empathy (CARE) score (for doctor consultations): aMD 1.2; 95% CI 0.28 to 2.13 Mean CARE score (for nurse consultations): aMD 1.11; 95% CI 0.03 to 2.19 Higher proportion of intervention patients were very satisfied with their care (42%) compared to those receiving usual care (39%) (MD 1.58, 95% CI 1.19 to 2.08, *p* = 0.0014). No significant differences in Self-rated health; Bayliss measure of illness burden; depression and anxiety (HAD scale); Treatment burden (MTBQ); Medication adherence (Moriskey measure) and number of medications; Number high risk prescriptions; Healthcare utilisation (GP and nurse visits, OPD visits and admissions) and Quality of care (QOF indicators) Cost-effectiveness: 50.8% chance of being cost-effective at a willingness-to-pay threshold of GBP20 000 per QALY (55.8% at £30 000 per QALY). Reported as ‘equivocal cost-effectiveness’
Schafer 2018 [41] Cluster RCT Germany (HRQoL data from author)	Primary outcomes: Number medications and HRQoL (EQ-5D) No difference group in the change of the number of medications taken: 0.43, 95% CI −0.07 to 0.93; *p* = 0.094 No difference in EQ-5D index score: aMD 0.03; 95% CI −0.03 to 0.09; *p* = 0.302.	Increase in prescribing of analgesics in the intervention group (Adjusted RR 2.043, *P* = 0.019) No significant differences in patient satisfaction; patient empowerment; depression; healthcare utilisation or in direct costs reported using Leipzig supply and Cost Instrument
Sommers 2000 [42] RCT USA	Primary outcome: Health service use Odds ratio admissions/patient/year 0.63 (95% CI 0.41 to 0.96); ≥ 1 60 day readmissions 0.26 (0.08 to 0.84). Not fully reported for seven other outcomes, non-significant for six. Difference in adjusted mean scores, social activities count 0.50 (95% CI 0.02 to 1.00). Symptom scale 0.50 (−3.20 to 0.16), SF-36 self-rated health 0.10 (−0.27 to 0.02), not reported for four other outcomes, non-significant	Social activities count: Int = 0.2 vs Con −0.3, *p* = 0.04 No significant differences in patient reported health status; social activities count; HRQoL (SF36); depression scores; nutrition checklists and drug adherence
**Self-management support studies**		
Eakin 2007 [28] RCT USA	Primary outcome: Dietary behaviour, and physical activity Adjusted means (SE): dietary behaviour (lower score better) 2.20 (0.05) *v* 2.41 (0.05), *p* < 0.5; change minutes walking/week 8 (22) *v* −10 (27), *p* > 0.5	Support for healthy lifestyle (higher score better) 2.98 (0.06) *v* 2.68 (0.06), *p* < 0.05
Garvey 2015 [29] RCT Ireland	Primary outcome: Activity participation Frenchay Activities Index aMD at immediate follow up 4.22, 95% CI 1.59 to 6.85.	Significant improvements in perceptions of activity performance and satisfaction, self-efficacy, independence in daily activities and HRQoL (EQ-5D VAS scores only). The intervention group demonstrated significantly higher levels of goal achievement, following the intervention. No significant differences in anxiety, depression, HeiQ scores or healthcare utilisation.
O’Toole 2019 [33] RCT Ireland (data from authors)	Primary outcomes: HRQoL (EQ5D) and Activity Participation (FAI) At 6-month follow-up there were no differences in primary outcomes: EQ5D index score aMD = 0.1; 95% CI −0.02 to 0.22 FAI aMD = 1.20; 95% CI −0.89 to 3.29	No significant difference in Activities of daily living (NEADL); Anxiety and depression (HADs); Self-efficacy and healthcare utilisation. One of the two occupational performance domains (COPM) showed a significant difference. There were two pre-planned sub-group analyses for the primary outcomes. There was no difference in effects by number of conditions but there was a significant improvement in the EQ5D VAS in those aged < 65 compared to those ≥ 65 years, a 23.13, 95% CI = 3.19 to 43.06, *p* = 0.0284.
Reed 2018 [34] RCT Australia	Primary outcome: Self-rated health intervention were more likely than control participants to report improved self-rated health at 6 months: Odds Ratio (2.50, 95% CI, 1.13 to 5.50, *p* = 0.023).	No significant differences in Fatigue; Pain; Health distress; Energy; Depression; Illness intrusiveness; Exercise; Medication adherence; Self-Efficacy; Health Education Impact (HEiQ); Healthcare utilisation (GP visits, Emergency Department (ED) visits and admissions)
**Medicines management studies**		
Jager 2017 [36] Cluster RCT Germany	Primary outcome: Summary score of 10 prescribing indicators The increase in the degree of implementation was 4.2 percentage points (95% CI −0.3 to 8.6) higher in the intervention group compared to the control group (*p* = 0.1).	Harms were not expected or reported No significant difference in Patient Activation Measure (PAM-13D); Medication Adherence Report Scale (MARS); Beliefs About Medicines Questionnaire (BMQ-D) and % Potentially Inappropriate Medicines (PIMs)
Koberlein Neu [37] cRCT 2016 (stepped wedge design) Germany	Primary outcome: Quality of medication therapy (mean MAI score) Mean MAI score: Intervention phase 1 vs Control Phase, aMD −4.51, 95% CI −6.66 to −2.36	Mean reduction in drug-related problems of −0.45, 95% CI −0.81 to −0.09 No significant difference in Number of drug-related problems (DRPs); Potentially inadequate medication (PIM); Number of prescribed medicines per patient; HRQoL (SF12); Function (Barthel Index); Instrumental Activities of Daily Living (IADL); Gait stability/risk of falling (Tinetti score) Level of social support results not reported.
Krska 2001 RCT [32] UK	Primary outcome: Pharmaceutical care issues. [outcome trial specific] Pharmaceutical care issues (%) resolved after intervention: 82.7% *v* 41.2%, *p* < 0.001	No significant differences in medicine costs, HRQoL (SF36 scores) and health service use
Muth 2018 [16] Cluster RCT Germany	Primary outcome: Medication Appropriateness Index (MAI) at 6 months No significant effect on mean MAI sum scores with aMD of 0.7 (95% CI −0.2 to 1.6)	Functional status (Vulnerable Elderly Survey-13) MD 0.4, 95% CI 0.0 to 0.8, *p* = 0.047 No significant difference in all other secondary outcomes including MAI at 9 months; HRQoL EQ-5D (aMD 2.3; 95% CI −1.6 to 6.2, *p* = 0.247); All-cause hospitalisation; Severity of chronic pain (von Korff Index); Satisfaction with shared decision-making (Man-Son-Hing Scale); Patient’s future expectation, expected/desired lifetime duration; Years of Desired Life (YDL); Medication adherence: Observed adherence: drug score, dose score, regimen score; Self-reported adherence (Morisky); Patient Beliefs about Medicines Questionnaire (BMQ); Medicines prescribed; Medication Regimen Complexity Index and number of prescriptions/single doses

**Fig. 3:**
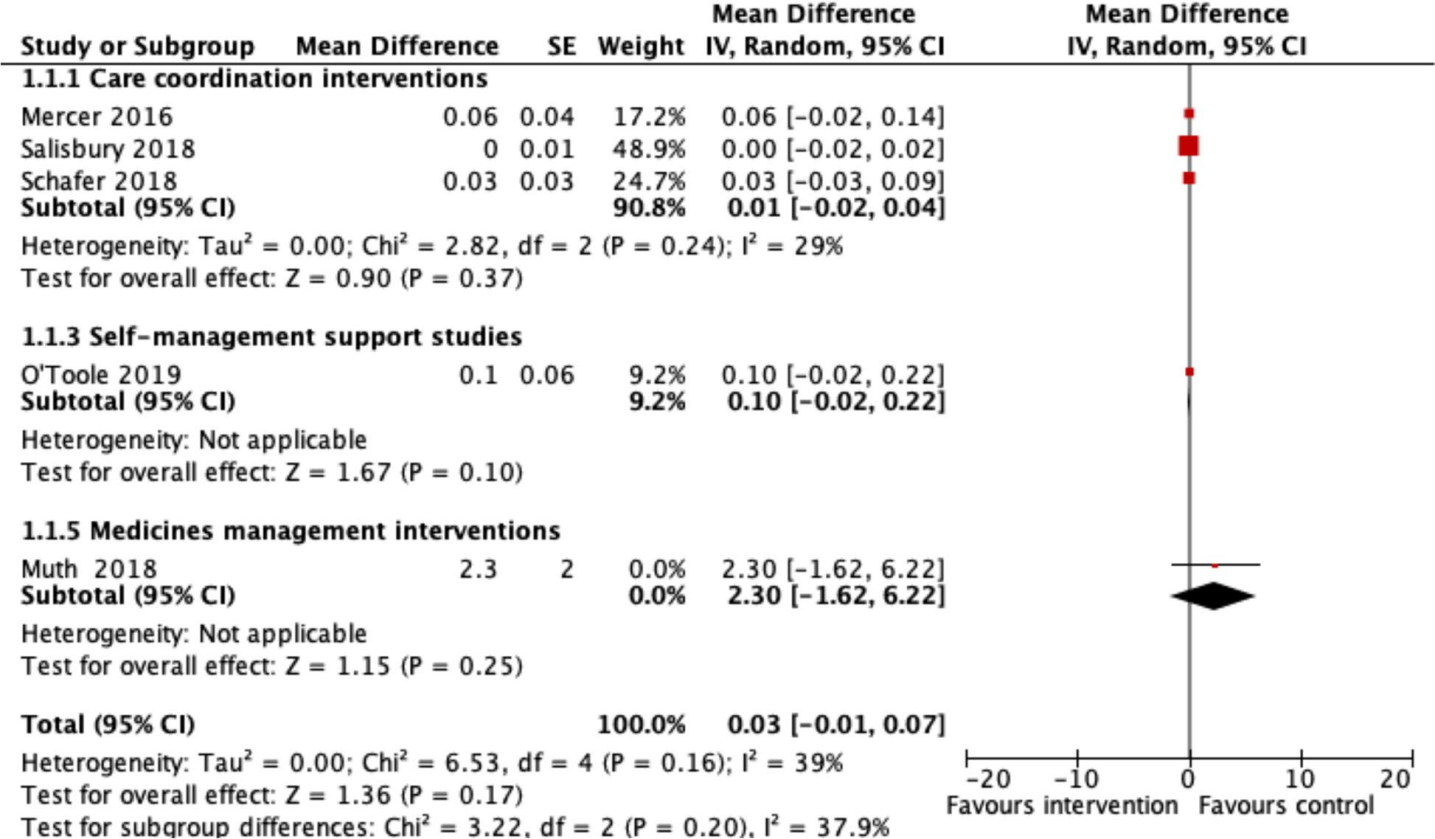
Meta-analysis of health-related quality of life (EQ-5D scores). Footnote: Estimates for all included cluster RCTs were adjusted for clustering

Overall, the results suggest that all intervention types targeting patients with multimorbidity probably make little or no difference to the main outcomes of HRQoL (*n* = 10) [29, 30, 32, 33, 37–42] or mental health outcomes (*n* = 6) [11, 29, 33, 40–42]. Five of the 10 studies with HRQoL outcomes reported EQ-5D scores that could be included in a meta-analysis [11, 33, 39–41], with a mean difference of 0.03 (95% CI −0.01 to 0.07, *I*^2^ = 39%) (see Fig. 3), consistent with the overall effect suggesting no difference in this outcome. The five studies included in the HRQoL meta-analysis had low risk of bias overall.

For additional outcomes, there was little or no effect on clinical outcomes (*n* = 2) or on the majority of other psychosocial outcomes (*n* = 11), including self-efficacy (*n* = 4). A meta-analysis of studies with available data for self-efficacy (*n* = 2) found a mean difference in self-efficacy scores of 0.92, 95% CI −0.04 to 1.88, *I*^2^ = 63% (see Additional file 4: Figure 2). There were mixed effects on function and activity (*n* = 4) and patient health behaviours (*n* = 2). There was little or no effect on healthcare utilisation (*n* = 9), though numbers of hospital admissions in most studies were small. There was also little or no effect on medicine outcomes (*n* = 9). Five of the care coordination/self-management support studies reported little or no effect on numbers of medicines or medication adherence. There were mixed effects on medicine outcomes in the four studies with a medicines management type intervention, which reported mixed results in medication appropriateness and potentially inappropriate prescribing. There was some improvement in healthcare provider behaviours in two of the three care coordination/self-management support studies reporting these outcomes but mixed effects on patient satisfaction with services (*n* = 3). Only one of the 16 included studies reported a potential adverse event relating to prescribing of analgesics but no other studies reported harms. Only two of the 16 studies reported full cost-effectiveness analyses to date with one reporting cost-effectiveness [38] and the other reporting equivocal results [40].

## Discussion

We identified 16 RCTs eligible for inclusion with a low risk of bias overall. The majority of studies included older patients with at least three conditions. Interventions were complex and multifaceted and could be broadly categorised into three groups involving care coordination and/or self-management support and medicines management. However, the heterogeneous populations and interventions make comparison of intervention effects difficult. Overall, despite 16 RCTs examining interventions for multimorbidity, there is still no clear high quality evidence to guide healthcare delivery with little effect on the main outcomes of health related quality of life or mental health outcomes. There was no clear pattern of effect by type of intervention. Care coordination/self-management support type interventions may improve the patient experience of care though this is based on a small number of studies and is of low certainty. Self-management support interventions may be associated with minimal improvements in patient health behaviours. Medicines management interventions had mixed effects but in some studies there may have been minimal room for improvement. However, these conclusions are based on small numbers of studies and are of low certainty.

The results suggest that future research for multimorbidity should consider areas such as the patient experience of care, optimising medicines management and targeted patient health behaviours such as exercise though this is based on a small numbers of studies and low certainty evidence. Twelve of the 16 included studies aimed to improve self-management support in patients. Many self-management support interventions are based on the original Chronic Disease Self-management Support Programme, and our results are consistent with the Cochrane Review on lay-led self-management support programmes, which concluded that whilst these interventions may have modest short-term effects on confidence to manage conditions, there is no clear evidence that these interventions improve psychological health, symptoms or health-related quality of life, or that they significantly alter healthcare use [43]. Addressing functional difficulties has been identified as a patient priority [15], but we found mixed effects on function and disability. Economic outcomes tended to focus on simple cost analyses comparing direct costs for intervention and control participants.

This review includes one of the largest studies undertaken in multimorbidity, the 3D study, which showed no difference in its primary outcome (HRQoL), despite having an intervention carefully designed to address the known challenges and treatment burden of multimorbidity and focusing on dimensions of health, depression and drugs (3D) [40]. However, the 3D intervention did improve patient-centred care, which may well be a reasonable end-point in itself [44]. One of the other larger multimorbidity studies, the Guided Care study, targeted high-risk older patients with multimorbidity, but found no overall effect on hospital admissions [35]. However, a pre-planned sub-group analysis indicated improvements in one of the participating healthcare organisations (Kaiser-Permanente, an insurance based care system in the USA, *n* = 365, 43% of full sample). Boult et al. postulated that this result may have been related to the fact that care was already more organised and structured in this system, so that the Guided Care intervention may simply have extended the existing approaches used in that setting, whereas its implementation was more challenging in less organised systems [35].

Even when interventions are targeted at a specific problem such as polypharmacy or potentially inappropriate prescribing, they may not be effective unless they target the right patients. For example, we found that, of the four studies with medicines management interventions, two included participants with minimal baseline prescribing problems making it difficult to improve outcomes. The Cochrane Review of interventions for enhancing medication adherence concluded that ‘current methods of improving adherence for chronic health problems are mostly complex and not very effective’ and suggests further research is needed [45]. Managing medicines is a key part of managing multimorbidity and features as a key element of existing clinical guidelines for multimorbidity with an emphasis on targeting those with more complex polypharmacy, i.e. on 15 or more regular medicines [13, 15, 16].

The majority of the studies in this review included older people, even when younger adults were eligible for inclusion. It is important to address the needs of younger individuals as there are additional issues to consider relating to employability and absenteeism. Individuals in the poorest socioeconomic groups are more likely to develop multimorbidity at a younger age [10]. This review includes a trial that specifically targeted socioeconomically disadvantaged people with multimorbidity [38]. This CarePlus study had a multi-level intervention supporting practitioners and patients and reported a cost per quality-adjusted life year of GBP 12,000 which is well within the recommended funding threshold for effective healthcare interventions in the UK.

The most consistent intervention element across all included studies was the use of case managers, but even these varied in that some were clinical case managers and others were administrative managers. Systematic reviews of community-based case management in general have indicated uncertain effects with improvements in client and professional satisfaction with care and reductions in caregiver strain, but no impact on healthcare utilisation [46]. An international group of multimorbidity researchers recently published a systematic review of clinical guidelines for multimorbidity and polypharmacy which also found variation in the eight clinical guidelines reviewed and a need for greater consensus on multimorbidity definitions and management approaches [16].

The largely negative findings in this review likely relates to the challenges of multimorbidity in terms of heterogeneity of populations and potential interventions. Whilst it could be argued that multimorbidity care may not offer any advantages over care for single chronic conditions, qualitative research with patients and practitioners highlights the challenges they face managing multiple conditions in medical systems that have largely been designed around single chronic condition care [47, 48]. The NICE Guidance on Multimorbidity calls for a re-orientation of care to address multimorbidity and highlights the importance of recognising and addressing treatment burden for patients [13, 14].

### Strengths and limitations of the review

Multimorbidity is a complex area because the characteristics of participants can vary depending on definitions used. This limits the potential to combine study results reasonably for meta-analysis. This clinical heterogeneity has led some to question whether defined interventions can be developed for this population. Despite this challenge, there are increasing numbers of interventions being developed and evaluated. The review was carried out using the updated *Cochrane Handbook for Systematic Reviews of Interventions* [49]. Potential limitations in the search process for this review related to the lack of a MeSH term for multimorbidity, though this has now been addressed. This meant that we originally had to use broad search terms which led to a high yield of citations to be searched. Given the very high number of titles screened this was done by only one author and this is a limitation of the review. However, the authors are active researchers in the field of multimorbidity and are unaware of any potentially eligible studies that were missed by the search. We were also unable to retrieve some missing data from authors. However, as limited meta-analyses were undertaken this did not lead to any appreciable measurement bias. The usual limitations relating to publication bias apply, but we have searched the grey literature and contacted experts in the field to try to identify published and ongoing trials in this area. A further limitation of the review is that the last full comprehensive search update was conducted in September 2019. In view of this limitation, we reviewed the comprehensive database of the International Research Community in Multimorbidity to review potential studies published between 2019 and 2021 [50]. Only two potentially eligible trials [51, 52] were identified and the results of these studies would not change the conclusions of the current systematic review.

The variation in definitions in the included studies included highlights the need for clear reporting of participant characteristics. Without these definitions, the generalisability or applicability of studies for people with multimorbidity will be uncertain [53]. When designing interventions, researchers need to be clear about the theoretical assumptions underlying the intervention, consider its individual components and the evidence base behind each, and then link these to outcomes. There is a specific framework to support the development of interventions for multimorbidity, which highlights the potential for other study designs, such as stepped-wedge designs that may be more suited to multimorbidity intervention initiatives and that can be undertaken within service/research partnerships [54]. There is also room to improve patient and public participation (PPI) in multimorbidity trials with only a few of the more recent studies in this review incorporating PPI [38, 40]. People with multimorbidity are more likely to experience what is referred to as ‘treatment burden’, that is, the effort needed to engage in the multiple treatments offered to them can actually make their lives more difficult [14]. Only one study included a treatment burden measure and reported little or no difference in this outcome [40]. Outcomes for this review were based on the core outcome set for multimorbidity, which can also inform outcome selection for future studies so that we can more easily compare interventions across different studies [26].

## Conclusion

This review highlights the growing evidence underpinning interventions to improve outcomes for people with multimorbidity. Despite the number of randomised controlled trials, there are remaining uncertainties about the effectiveness of interventions for people with multimorbidity. Our findings suggest that future research for multimorbidity should consider areas such as the patient experience of care, optimising medicines management and targeting patient health behaviours such as exercise. There are significant numbers of ongoing multimorbidity studies, all of which will generate much needed further evidence to support the development of healthcare services to improve outcomes for patients with multimorbidity.

## Supplementary Information

**Additional file 1.** Search Strategies.

**Additional file 2: Figure 1.** Risk of bias in included studies.

**Additional file 3.** Grade Working sheets.

**Additional file 4: Figure 2.** Meta-analysis of self-efficacy scores.

## Data Availability

Data available for the authors.
