# Supplementary material for: Interventions for improving outcomes in patients with multimorbidity in primary care and community setting: a systematic review
Source: Syst Rev. 2021 Oct 20;10:271. doi: 10.1186/s13643-021-01817-z (PMC8527775; doi:10.1186/s13643-021-01817-z)
Supplement: Supplementary file 1 — Additional file 1. Search Strategies. [file 13643_2021_1817_MOESM1_ESM.docx]

**Additional file 1: Search Strategies**

**Medline (OVID)**

| No. | Search terms | Results |
| --- | --- | --- |
| 1 | comorbidity/ | 93683 |
| 2 | multimorbidity/ | 142 |
| 3 | chronic disease/ | 248267 |
| 4 | (comorbid* or comorbid*).ti,ab,kf. | 142140 |
| 5 | (multimorbid* or multi-morbid*).ti,ab,kf. | 3780 |
| 6 | (multidisease? or multicondition? or ((multi or multiple) adj2 (morbid* or ill* or disease? or condition? or syndrom* or disorder?))).ti,ab,kf. | 32175 |
| 7 | (chronic* adj (disease? or ill* or care or condition? or disorder* or health* or medication* or syndrom* or symptom*)).ti,ab,kf. | 97771 |
| 8 | or/1-7 | 539843 |
| 9 | exp primary health care/ | 137724 |
| 10 | family practice/ | 63867 |
| 11 | physicians, primary care/ | 2607 |
| 12 | general practice/ | 11472 |
| 13 | physicians, family/ | 15795 |
| 14 | general practitioners/ | 6162 |
| 15 | exp outpatient clinics, hospital/ | 16562 |
| 16 | ambulatory care/ | 40221 |
| 17 | exp ambulatory care facilities/ | 51562 |
| 18 | exp community health services/ | 279963 |
| 19 | exp community health centers/ | 11678 |
| 20 | ((primary or communit*) adj5 (care or health*)).ti,ab,kf. | 210782 |
| 21 | (family practi* or family doctor* or family physician* or gp* or general practi*).ti,ab,kf. | 240907 |
| 22 | ((outpatient? or ambulatory) adj2 (care or healthcare or clinic? or service? or facilit*)).ti,ab,kf. | 54859 |
| 23 | (community adj2 (site? or practice? or clinic? or based or facilit*)).ti,ab,kf. | 62174 |
| 24 | or/9-23 | 897153 |
| 25 | ((organisation* or organization*) adj2 (intervention* or program*)).ti,ab,kf. | 2194 |
| 26 | (self care or self management).ti,ab,kf. | 28575 |
| 27 | (case management or care management).ti,ab,kf. | 16604 |
| 28 | ((integrat* or coordinated or co-ordinated or collaborat*) adj2 care).ti,ab,kf. | 13646 |
| 29 | ((financ* or money or monetary or cash) adj2 incentiv*).ti,ab,kf. | 5136 |
| 30 | (patient adj2 educat*).ti,ab,kf. | 19233 |
| 31 | ((provider? or physician? or doctor? or nurse or health or healthcare) adj2 educat*).ti,ab,kf. | 59505 |
| 32 | ((multicomponent or multi-component or multifacet* or multi-facet* or multidisciplinary or multi-disciplinary or interdisciplinary or inter-disciplinary) adj2 (care or team? or intervention? or program*)).ti,ab,kf. | 32379 |
| 33 | (care plan? or guided care or (personal* adj care)).ti,ab,kf. | 12297 |
| 34 | (home adj2 (care or intervention or program)).ti,ab,kf. | 26996 |
| 35 | exp self care/ | 49774 |
| 36 | self-management/ | 525 |
| 37 | case management/ | 9508 |
| 38 | case managers/ | 55 |
| 39 | "delivery of health care, integrated"/ | 10901 |
| 40 | patient care planning/ | 37134 |
| 41 | exp insurance, health, reimbursement/ | 43219 |
| 42 | patient care team/ | 60477 |
| 43 | home care services/ | 31238 |
| 44 | patient education as topic/ | 79695 |
| 45 | exp education professional/ | 275487 |
| 46 | exp inservice training/ | 27351 |
| 47 | or/25-46 | 708583 |
| 48 | randomised controlled trial.pt. | 462301 |
| 49 | controlled clinical trial.pt. | 92438 |
| 50 | multicenter study.pt. | 234567 |
| 51 | pragmatic clinical trial.pt. | 791 |
| 52 | (randomis* or randomiz* or randomly).ti,ab. | 774282 |
| 53 | groups.ab. | 1800888 |
| 54 | (trial or multicenter or multi center or multicentre or multi centre).ti. | 216690 |
| 55 | (intervention? or effect? or impact? or controlled or control group? or (before adj5 after) or (pre adj5 post) or ((pretest or pre test) and (posttest or post test)) or quasiexperiment* or quasi experiment* or pseudo experiment* or pseudoexperiment* or evaluat* or time series or time point? or repeated measur*).ti,ab. | 8468868 |
| 56 | non-randomised controlled trials as topic/ | 356 |
| 57 | interrupted time series analysis/ | 436 |
| 58 | controlled before-after studies/ | 327 |
| 59 | or/48-58 | 9453070 |
| 60 | exp animals/ | 21566819 |
| 61 | humans/ | 17103658 |
| 62 | 60 not (60 and 61) | 4463161 |
| 63 | review.pt. | 2386895 |
| 64 | meta analysis.pt. | 89092 |
| 65 | news.pt. | 188085 |
| 66 | comment.pt. | 718043 |
| 67 | editorial.pt. | 458787 |
| 68 | cochrane database of systematic reviews.jn. | 13619 |
| 69 | comment on.cm. | 718039 |
| 70 | (systematic review or literature review).ti. | 112762 |
| 71 | or/62-70 | 7910709 |
| 72 | 59 not 71 | 6617220 |
| 73 | 8 and 24 and 47 and 72 | 6414 |

**Embase (OVID)**

| No. | Search terms | Results |
| --- | --- | --- |
| 1 | comorbidity/ | 207222 |
| 2 | multiple chronic conditions/ | 812 |
| 3 | chronic disease/ | 179584 |
| 4 | (comorbid* or comorbid*).ti,ab,kw. | 250506 |
| 5 | (multimorbid* or multi-morbid*).ti,ab,kw. | 5038 |
| 6 | (multidisease? or multicondition? or ((multi or multiple) adj2 (morbid* or ill* or disease? or condition? or syndrom* or disorder?))).ti,ab,kw. | 48176 |
| 7 | (chronic* adj (disease? or ill* or care or condition? or disorder* or health* or medication* or syndrom* or symptom*)).ti,ab,kw. | 134309 |
| 8 | or/1-7 | 625986 |
| 9 | exp primary health care/ | 146151 |
| 10 | general practice/ | 78166 |
| 11 | general practitioner/ | 86251 |
| 12 | outpatient department/ | 57351 |
| 13 | outpatient care/ | 31544 |
| 14 | ambulatory care/ | 35397 |
| 15 | community care/ | 55612 |
| 16 | ((primary or communit*) adj5 (care or health*)).ti,ab,kw. | 267647 |
| 17 | (family practi* or family doctor* or family physician* or gp* or general practi*).ti,ab,kw. | 309049 |
| 18 | ((outpatient? or ambulatory) adj2 (care or healthcare or clinic? or service? or facilit*)).ti,ab,kw. | 83905 |
| 19 | (community adj2 (site? or practice? or clinic? or based or facilit*)).ti,ab,kw. | 79352 |
| 20 | or/9-19 | 855854 |
| 21 | ((organisation* or organization*) adj2 (intervention* or program*)).ti,ab,kw. | 3055 |
| 22 | (self care or self management).ti,ab,kw. | 40162 |
| 23 | (case management or care management).ti,ab,kw. | 21641 |
| 24 | ((integrat* or coordinated or co-ordinated or collaborat*) adj2 care).ti,ab,kw. | 18758 |
| 25 | ((financ* or money or monetary or cash) adj2 incentiv*).ti,ab,kw. | 6363 |
| 26 | (patient adj2 educat*).ti,ab,kw. | 29772 |
| 27 | ((provider? or physician? or doctor? or nurse or health or healthcare) adj2 educat*).ti,ab,kw. | 69095 |
| 28 | ((multicomponent or multi-component or multifacet* or multi-facet* or multidisciplinary or multi-disciplinary or interdisciplinary or inter-disciplinary) adj2 (care or team? or intervention? or program*)).ti,ab,kw. | 54100 |
| 29 | (care plan? or guided care or (personal* adj care)).ti,ab,kw. | 16871 |
| 30 | (home adj2 (care or intervention or program)).ti,ab,kw. | 33134 |
| 31 | exp self care/ | 69679 |
| 32 | case management/ | 10378 |
| 33 | case manager/ | 1384 |
| 34 | integrated health care system/ | 9728 |
| 35 | patient care planning/ | 29229 |
| 36 | reimbursement/ | 50415 |
| 37 | exp home care/ | 66904 |
| 38 | patient education/ | 103716 |
| 39 | education program/ | 45985 |
| 40 | in service training/ | 15552 |
| 41 | continuing education/ | 30265 |
| 42 | vocational education/ | 9758 |
| 43 | or/21-42 | 586250 |
| 44 | randomised controlled trial/ | 503050 |
| 45 | controlled clinical trial/ | 461463 |
| 46 | quasi experimental study/ | 4588 |
| 47 | pretest posttest control group design/ | 342 |
| 48 | time series analysis/ | 20787 |
| 49 | experimental design/ | 15488 |
| 50 | multicenter study/ | 185670 |
| 51 | (randomis* or randomiz* or randomly).ti,ab. | 1068299 |
| 52 | groups.ab. | 2449822 |
| 53 | (trial or multicentre or multicenter or multi centre or multi center).ti. | 301367 |
| 54 | (intervention? or effect? or impact? or controlled or control group? or (before adj5 after) or (pre adj5 post) or ((pretest or pre test) and (posttest or post test)) or quasiexperiment* or quasi experiment* or pseudo experiment* or pseudoexperiment* or evaluat* or time series or time point? or repeated measur*).ti,ab. | 10816607 |
| 55 | or/44-54 | 12064796 |
| 56 | (systematic review or literature review).ti. | 132462 |
| 57 | "cochrane database of systematic reviews".jn. | 11854 |
| 58 | exp animals/ or exp invertebrate/ or animal experiment/ or animal model/ or animal tissue/ or animal cell/ or nonhuman/ | 26063529 |
| 59 | human/ or normal human/ or human cell/ | 19717589 |
| 60 | 58 not (58 and 59) | 6394318 |
| 61 | 56 or 57 or 60 | 6537365 |
| 62 | 55 not 61 | 9208470 |
| 63 | 8 and 20 and 43 and 62 | 6986 |

**The Cochrane Library**

| No. | Search terms | Results |
| --- | --- | --- |
| #1 | [mh comorbidity] | 3624 |
| #2 | [mh multimorbidity] | 10 |
| #3 | [mh "chronic disease"] | 12784 |
| #4 | (comorbid* or comorbid*):ti,ab | 11761 |
| #5 | (multimorbid* or multi-morbid*):ti,ab | 200 |
| #6 | (multidisease? or multicondition? or ((multi or multiple) near/2 (morbid* or ill* or disease? or condition? or syndrom* or disorder?))):ti,ab | 691 |
| #7 | (chronic* next (disease? or ill* or care or condition? or disorder* or health* or medication* or syndrom* or symptom*)):ti,ab | 4865 |
| #8 | ^1-#7^ | 31367 |
| #9 | [mh "primary health care"] | 7397 |
| #10 | [mh "family practice"] | 2215 |
| #11 | [mh "physicians, primary care"] | 147 |
| #12 | [mh "general practice"] | 2636 |
| #13 | [mh "physicians, family"] | 488 |
| #14 | [mh "general practitioners"] | 220 |
| #15 | [mh "outpatient clinics, hospital"] | 721 |
| #16 | [mh "ambulatory care"] | 3897 |
| #17 | [mh "ambulatory care facilities"] | 1965 |
| #18 | [mh "community health services"] | 13906 |
| #19 | [mh "community health centers"] | 595 |
| #20 | ((primary or communit*) near/5 (care or health*)):ti,ab | 22580 |
| #21 | (family next practi* or family next doctor* or family next physician* or gp* or general next practi*):ti,ab | 13356 |
| #22 | ((outpatient? or ambulatory) near/2 (care or healthcare or clinic? or service? or facilit*)):ti,ab | 824 |
| #23 | (community near/2 (site? or practice? or clinic? or based or facilit*)):ti,ab | 6388 |
| #24 | ^15-#23^ | 54355 |
| #25 | ((organisation* or organization*) near/2 (intervention* or program*)):ti,ab | 356 |
| #26 | (self next care or self next management):ti,ab | 6001 |
| #27 | (case next management or care next management):ti,ab | 2251 |
| #28 | ((integrat* or coordinated or co-ordinated or collaborat*) near/2 care):ti,ab | 1545 |
| #29 | ((financ* or money or monetary or cash) near/2 incentiv*):ti,ab | 844 |
| #30 | (patient near/2 educat*):ti,ab | 2298 |
| #31 | ((provider? or physician? or doctor? or nurse or health or healthcare) near/2 educat*):ti,ab | 3936 |
| #32 | ((multicomponent or multi-component or multifacet* or multi-facet* or multidisciplinary or multi-disciplinary or interdisciplinary or inter-disciplinary) near/2 (care or team? or intervention? or program*)):ti,ab | 1669 |
| #33 | (care next plan? or guided next care or (personal* next care)):ti,ab | 459 |
| #34 | (home near/2 (care or intervention or program)):ti,ab | 3320 |
| #35 | [mh "self care"] | 5543 |
| #36 | [mh self-management] | 67 |
| #37 | [mh "case management"] | 812 |
| #38 | [mh "case managers"] | 5 |
| #39 | [mh "delivery of health care, integrated"] | 400 |
| #40 | [mh "patient care planning"] | 1858 |
| #41 | [mh "insurance, health, reimbursement"] | 496 |
| #42 | [mh "patient care team"] | 1839 |
| #43 | [mh "home care services"] | 2679 |
| #44 | [mh "patient education as topic"] | 8622 |
| #45 | [mh "education professional"] | 4409 |
| #46 | [mh "inservice training"] | 781 |
| #47 | ^26-#46^ | 37828 |
| #48 | #8 and #24 and #47 | 1359 |

**CINAHL (EBSCO)**

| No. | Search terms | Results |
| --- | --- | --- |
| S1 | (MH "Comorbidity") | 30,004 |
| S2 | (MH "Chronic Disease") | 36,025 |
| S3 | (comorbid* or comorbid*) | 48,319 |
| S4 | (multimorbid* or multi-morbid*) | 806 |
| S5 | (multidisease? or multicondition? or ((multi or multiple) N2 (morbid* or ill* or disease? or condition? or syndrom* or disorder?))) | 4,857 |
| S6 | (chronic* N0 (disease? or ill* or care or condition? or disorder* or health* or medication* or syndrom* or symptom*)) | 26,406 |
| S7 | S1 OR S2 OR S3 OR S4 OR S5 OR S6 | 103,093 |
| S8 | (MH "Primary Health Care") | 38,036 |
| S9 | (MH "Family Practice") | 12,956 |
| S10 | (MH "Physicians, Family") | 10,411 |
| S11 | (MH "Ambulatory Care Facilities") | 4,109 |
| S12 | (MH "Outpatient Service") | 4,506 |
| S13 | (MH "Ambulatory Care") | 7,188 |
| S14 | (MH "Community Health Services+") | 279,888 |
| S15 | (MH "Community Health Centers+") | 3,782 |
| S16 | (primary or communit*) N5 (care or health*) | 134,831 |
| S17 | family practi* or family doctor* or family physician* or gp* or general practi* | 48,187 |
| S18 | (outpatient? or ambulatory) N2 (care or healthcare or clinic? or service? or facilit*) | 15,132 |
| S19 | (community N2 (site? or practice? or clinic? or based or facilit*)) | 22,651 |
| S20 | S8 OR S9 OR S10 OR S11 OR S12 OR S13 OR S14 OR S15 OR S16 OR S17 OR S18 OR S19 | 408,330 |
| S21 | ((organisation* or organization*) N2 (intervention* or program*)) | 1,484 |
| S22 | (self care or self management) | 37,953 |
| S23 | (case management or care management) | 33,785 |
| S24 | ((integrat* or coordinated or co-ordinated or collaborat*) N2 care) | 14,558 |
| S25 | ((financ* or money or monetary or cash) N2 incentiv*) | 1,637 |
| S26 | (patient N2 educat*) | 57,496 |
| S27 | ((provider? or physician? or doctor? or nurse or health or healthcare) N2 educat*) | 75,285 |
| S28 | ((multicomponent or multi-component or multifacet* or multi-facet* or multidisciplinary or multi-disciplinary or interdisciplinary or inter-disciplinary) N2 (care or team? or intervention? or program*)) | 32,872 |
| S29 | (care plan? or guided care or (personal* care)) | 14,685 |
| S30 | (home N2 (care or intervention or program)) | 38,054 |
| S31 | (MH "Self Care+") | 32,019 |
| S32 | (MH "Case Management") | 13,312 |
| S33 | (MH "Case Managers") | 2,797 |
| S34 | (MH "Health Care Delivery, Integrated") | 6,356 |
| S35 | (MH "Multidisciplinary Care Team") | 28,129 |
| S36 | (MH "Patient Care Plans+") | 6,753 |
| S37 | (MH "Insurance, Health, Reimbursement+") | 26,500 |
| S38 | (MH "Home Health Care+") | 34,920 |
| S39 | (MH "Patient Education+") | 55,088 |
| S40 | (MH "Education+") | 593,604 |
| S41 | S21 OR S22 OR S23 OR S24 OR S25 OR S26 OR S27 OR S28 OR S29 OR S30 OR S31 OR S32 OR S33 OR S34 OR S35 OR S36 OR S37 OR S38 OR S39 OR S40 | 762,001 |
| S42 | S7 AND S20 AND S41 | 9,603 |
| S43 | PT randomised controlled trial | 44,643 |
| S44 | PT clinical trial | 55,952 |
| S45 | PT research | 1,207,634 |
| S46 | (MH "Randomised Controlled Trials") | 42,160 |
| S47 | (MH "Clinical Trials") | 93,247 |
| S48 | (MH "Intervention Trials") | 7,060 |
| S49 | (MH "Nonrandomised Trials") | 274 |
| S50 | (MH "Experimental Studies") | 17,888 |
| S51 | (MH "Pretest-Posttest Design+") | 31,833 |
| S52 | (MH "Quasi-Experimental Studies+") | 10,664 |
| S53 | (MH "Multicenter Studies") | 36,342 |
| S54 | (MH "Health Services Research") | 8,110 |
| S55 | TI ( randomis* or randomiz* or randomly) OR AB ( randomis* or randomiz* or randomly) | 143,792 |
| S56 | TI (trial or effect* or impact* or intervention* or before N5 after or pre N5 post or ((pretest or "pre test") and (posttest or "post test")) or quasiexperiment* or quasi W0 experiment* or pseudo experiment* or pseudoexperiment* or evaluat* or "time series" or time W0 point* or repeated W0 measur*) OR AB (trial or effect* or impact* or intervention* or before N5 after or pre N5 post or ((pretest or "pre test") and (posttest or "post test")) or quasiexperiment* or quasi W0 experiment* or pseudo experiment* or pseudoexperiment* or evaluat* or "time series" or time W0 point* or repeated W0 measur*) | 985,244 |
| S57 | S43 OR S44 OR S45 OR S46 OR S47 OR S48 OR S49 OR S50 OR S51 OR S52 OR S53 OR S54 OR S55 OR S56 | 1,599,424 |
| S58 | S42 AND S57 | 6,656 |
| S59 | S58 Limiters - Exclude MEDLINE records | 2,400 |

**ClinicalTrials.gov**

multimorbidity OR comorbidity

**WHO International Clinical Trials Registry Platform (ICTRP)**

multimorbidity OR comorbidity
