# Supplementary figures and images for: Interventions for improving outcomes in patients with multimorbidity in primary care and community setting: a systematic review
Source: Syst Rev. 2021 Oct 20;10:271. doi: 10.1186/s13643-021-01817-z (PMC8527775; doi:10.1186/s13643-021-01817-z)

**Additional file 2: Figure 1 : Risk of bias in included studies**


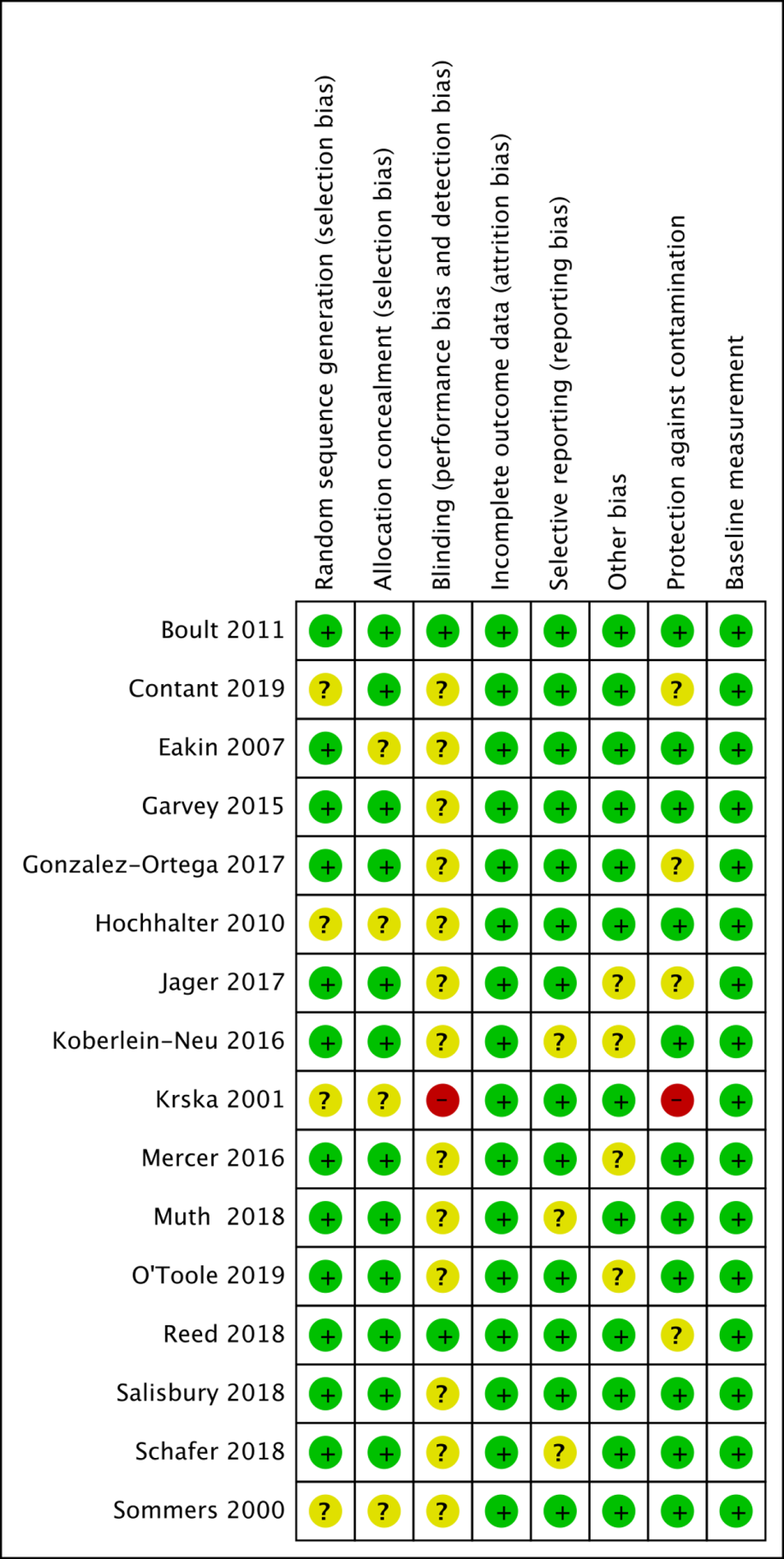

Supplement: Supplementary file 2 — Additional file 2: Figure 1. Risk of bias in included studies. [file 13643_2021_1817_MOESM2_ESM.docx]

**Additional file 4: Figure 2. Meta-analysis of self-efficacy scores**


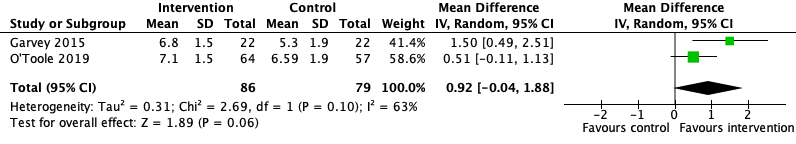

Supplement: Supplementary file 4 — Additional file 4: Figure 2. Meta-analysis of self-efficacy scores. [file 13643_2021_1817_MOESM4_ESM.docx]
