# Supplementary material for: Interventions for improving outcomes in patients with multimorbidity in primary care and community setting: a systematic review
Source: Syst Rev. 2021 Oct 20;10:271. doi: 10.1186/s13643-021-01817-z (PMC8527775; doi:10.1186/s13643-021-01817-z)
Supplement: Supplementary file 3 — Additional file 3. Grade Working sheets. [file 13643_2021_1817_MOESM3_ESM.docx]

**Additional file 3: Grade Working sheets**

| **Interventions aimed at improving outcomes for people with multimorbidity compared with usual care** |
| --- |
| **Assessed by:** SMS and BC |
| **Date: October 2020** |

**Worksheet: Assessing the certainty**^[[1]](#footnote-1)^ **of evidence across studies for an outcome**

**Comparison: Usual Care**

***Certainty assessment of evidence for each outcome***

| **No of studies** | **Design** | **Risk of bias** | **Inconsistency** | **Indirectness^^[[2]](#footnote-2)^^** | **Imprecision** | **Other^^[[3]](#footnote-3)^^** | **Certainty**  **(overall score)^^[[4]](#footnote-4)^^** |
| --- | --- | --- | --- | --- | --- | --- | --- |
| **Main outcomes:**  **Health Related Quality of Life** | | | | | | | |
| 8 | RCTs | Not serious | Serious | Not serious | Serious |  | Downgraded to Low due to inconsistency and imprecision |
| **Mental Health** | | | | | | | |
| 7 | RCTs | Not serious | Serious | Not serious | Serious |  | Downgraded to Low due to inconsistency and imprecision |
| **Other outcomes** | | | | | | | |
| **Clinical Outcomes** | | | | | | | |
| 2 | RCTs | Not serious | Serious | Not serious | Serious |  | Downgraded to Very Low due to inconsistency, indirectness and imprecision |
| **Other psychosocial outcomes including self-efficacy and function** | | | | | | | |
| 11 | RCTs | Not serious | Serious | Not serious | Serious |  | Downgraded to Low due to inconsistency and imprecision |
| **Patient health behaviours** | | | | | | | |
| 2 | RCTs | Not serious | Serious | Not serious | Serious |  | Downgraded to Low due to inconsistency and imprecision |
| **Health Service Utilisation** | | | | | | | |
| 9 | RCTs | Not serious | Serious | Not serious | Serious |  | Downgraded to Low due to inconsistency and imprecision |
| **Medicines outcomes** | | | | | | | |
| 9 | RCTs | Not serious | Serious | Not serious | Serious |  | Downgraded to Low due to inconsistency and imprecision |
| **Provider Behaviour** | | | | | | | |
| 4 | RCTs | Not serious | Serious | Not serious | Serious |  | Downgraded to Low due to inconsistency and imprecision |

**Detailed description**

- Risk of bias was generally low across all studies so not judged to be a serious concern
- Inconsistency: Studies for each outcome generally showed little or no effect and even when some studies showed possible effects, others within that comparison were not consistent for that outcome and differences between studies targeting co-morbidity compared to multimorbidity suggesting inconsistency in the overall body of evidence, so downgraded by 1.
- Indirectness – included studies are directly related to our research question (PICO) as it was very broad and allowed for inclusion of both multimorbidity and comorbidity and different age groups. This applied to clinical outcomes which were downgraded by 1 on this basis.

Imprecision was judged using the narrative synthesis of results and the meta-analyses and the meta-analyses only included limited numbers of studies for each comparison, so downgraded by 1

1. This can also be referred to as ‘quality of the evidence’ or ‘confidence in the estimate’. The “certainty of the evidence” is an assessment of how good an indication the research provides of the likely effect; i.e. the likelihood that the effect will be substantially different from what the research found. By “substantially different” we mean a large enough difference that it might affect a decision. [↑](#footnote-ref-1)
2. Indirectness includes consideration of

   - Indirect (between study) comparisons
   - Indirect (surrogate) outcomes
   - Applicability (study populations, interventions or comparisons that are different than those of interest)

   [↑](#footnote-ref-2)
3. Other considerations for downgrading include publication bias. Other considerations for upgrading include a strong association with no plausible confounders, a dose response relationship, and if all plausible confounders or biases would decrease the size of the effect (if there is evidence of an effect), or increase it if there is evidence of no harmful effect (safety) [↑](#footnote-ref-3)
4. 4
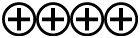
 **High** = This research provides a very good indication of the likely effect. The likelihood that the effect will be substantially different** is low.

   3
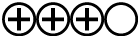
 **Moderate** = This research provides a good indication of the likely effect. The likelihood that the effect will be substantially different** is moderate.

   2
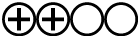
 **Low** = This research provides some indication of the likely effect. However, the likelihood that it will be substantially different** is high.

   1
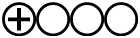
 **Very low** = This research does not provide a reliable indication of the likely effect. The likelihood that the effect will be substantially different** is very high.

   ** Substantially different = a large enough difference that it might affect a decision [↑](#footnote-ref-4)
